# Highly Pathogenic H5N1 Avian Influenza: Entry Pathways into North America via Bird Migration

**DOI:** 10.1371/journal.pone.0000261

**Published:** 2007-02-28

**Authors:** A. Townsend Peterson, Brett W. Benz, Monica Papeş

**Affiliations:** Natural History Museum and Biodiversity Research Center, The University of Kansas, Lawrence, Kansas, United States of America; US Naval Medical Research Center Detachment/Centers for Disease Control, United States of America

## Abstract

Given the possibility of highly pathogenic H5N1 avian influenza arriving in North America and monitoring programs that have been established to detect and track it, we review intercontinental movements of birds. We divided 157 bird species showing regular intercontinental movements into four groups based on patterns of movement—one of these groups (breed Holarctic, winter Eurasia) fits well with the design of the monitoring programs (i.e., western Alaska), but the other groups have quite different movement patterns, which would suggest the importance of H5N1 monitoring along the Pacific, Atlantic, and Gulf coasts of North America.

## Introduction

Flu viruses have long been known to circulate in birds [1], which led to intensive studies of avian influenzas in the middle twentieth century [2], [3]. The emergence of a highly pathogenic influenza A strain H5N1 in the past several years—beginning in Southeast Asia and now spreading into Africa, the Middle East, and Europe [1], [4]–[9]—has brought this issue again to the forefront of global attention. The fear is a global influenza pandemic—this highly pathogenic H5N1 strain (hereafter referred to as “HP-H5N1”) is associated with human case fatality rates reportedly higher than in the 1918 “Spanish” flu pandemic, and lacks only the ability to be transmitted efficiently among humans [10]–[12].

HP-H5N1, however, is for the moment, at least, an avian phenomenon—that is, although it is dangerous to humans once they are infected, HP-H5N1 is a bird disease transmitted among birds. If, at some point, HP-H5N1 evolves efficient human-to-human transmissability, then human movements and connectivity will govern the spread of the virus [13]. Until that time, however, HP-H5N1 is being dispersed by birds, be they wild or domestic. Although the issue of the relative importance of the two has seen considerable debate [14]–[19], and will clearly see considerably more debate still, we present herein a series of forecasts of likely HP-H5N1 geographic behavior regarding entry into North America *if dispersal were to be* driven by migratory birds.

### HP-H5N1 spread

HP-H5N1 was first isolated in 1996 from a farm goose in Guangdong Province, People's Republic of China [20]. It was then detected in domestic poultry and humans in Hong Kong in 1997. After a silent period, HP-H5N1 reappeared in quick succession in Thailand (Dec. 2003), Republic of Korea (Dec. 2003), Vietnam (Jan. 2004), Japan (Jan. 2004), Thailand (Jan. 2004), Cambodia (Jan. 2004), Laos People's Democratic Republic (Jan. 2004), Indonesia (Feb. 2004), and People's Republic of China (Feb. 2004). The next set of appearances began with records in People's Republic of China, Indonesia, Thailand, and Vietnam in June and July of 2004, and then in Malaysia (Aug. 2004).

After somewhat of a pause late in 2004, interesting events began to occur. A large outbreak (6000+ birds dying) occurred at Qinghai Lake in central China (Apr. 2005), as well as in poultry in Xinjiang Autonomous Region, western China (Jun. 2005), both well to the northwest of previous detections. In quick succession, outbreaks then occurred in western Siberia (Russia, Jul. 2005), Kazakhstan (Aug. 2005), Tibet (Aug. 2005), and Mongolia (Aug. 2005). Finally, late in 2005, HP-H5N1 appeared in Turkey, Romania, Taiwan, and Croatia (all in Oct. 2005), and later (late 2005–early 2006) in Cyprus, Saudi Arabia, Kuwait, and Iraq. As of April 2006, the virus had been detected across almost all of Europe, in numerous southern Asian countries, as well as in 5 African countries (Burkina Faso, Cameroon, Egypt, Nigeria, Niger), and by October 2006 the list had expanded to include Sudan, Ivory Coast, and others.

To summarize, from an initial southeastern appearance in Asia, HP-H5N1 spread up the Pacific coast as far as Japan and Korea. It later jumped northward into central Asia, and then appeared to the southwest in the Middle East, Europe, and Africa. These “jumps” have lead many to expect increasingly rapid spread, perhaps even globally, in coming years. In reality, though, we suspect that the apparently *extremely* rapid spread in recent years is most likely a reflection of establishment of surveillance programs—the virus had already spread to most of the areas listed above prior to initiation of surveillance efforts.

That wild birds are an important long-term evolutionary source reservoir of influenza A viruses is in little doubt [1]. However, for HP-H5N1 in particular, the pattern of spread has been variably interpreted as reflecting involvement of wild birds [21]–[23] or *not* being at all consistent with wild bird movements [14], [15], [17], [24]. The basic argument revolves around the relative weights of evidence including where the virus has and has not been documented to occur, and whether the virus circulates among healthy wild birds. In this contribution, however, we reconstruct likely HP-H5N1 geographic points of entry from Eurasia and Africa into North America under the supposition that wild birds are involved, summarizing migratory patterns of 157 bird species that have distributional areas in both hemispheres.

## Results

**Figure 1:**
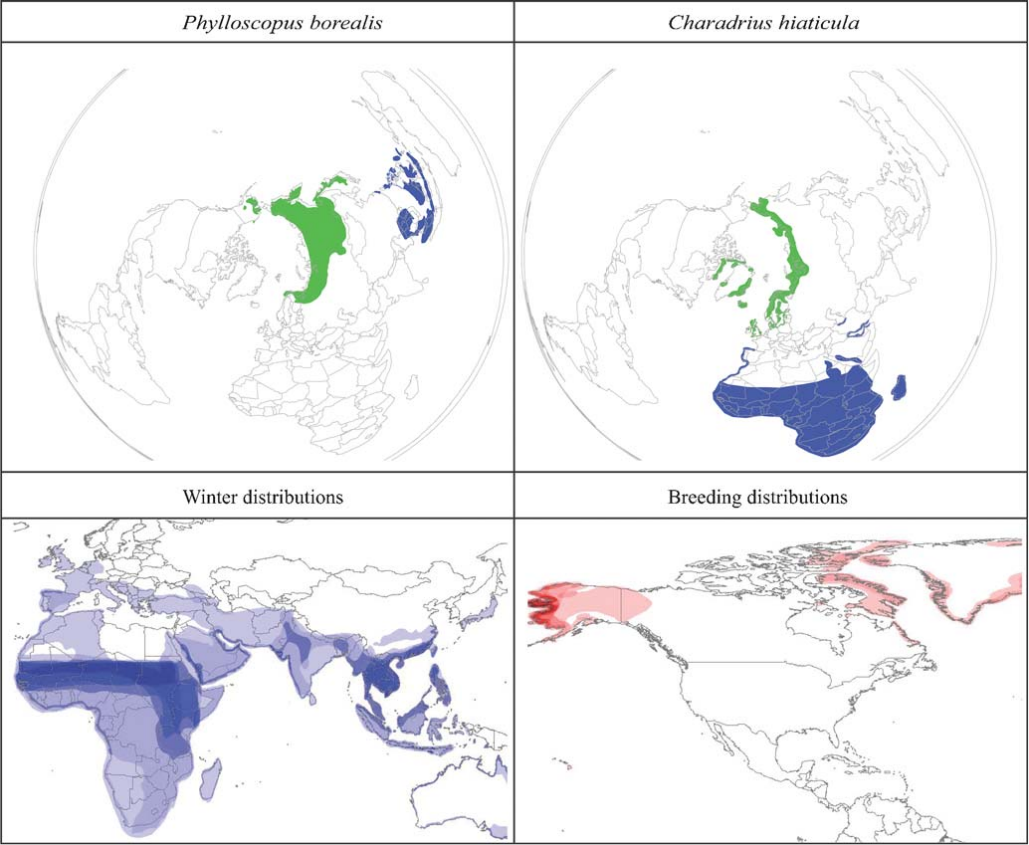
Species that winter in Eurasia, but that breed across the Holarctic (including North America). The top 2 panels present examples of single species (Phylloscopus borealis, Charadrius hiaticula; green = breeding distribution, blue = wintering distribution), whereas the lower 2 panels show species richness patterns of this set of species (see Appendix S1) in winter (white = 0, darkest shade = 8 species) and in the breeding season (white = 0, darkest shade = 11 species), with color ramp indicating number of species.

In all, we could divide the 157 species more or less concretely into 4 groups (lists provided in Appendix S1). The traditional group considered in the context of HP-H5N1 were the 14 species that winter in Eurasia, but that breed across the Holarctic (i.e., crossing into the Western Hemisphere at least marginally; Fig. 1). These species winter across West and Central Africa, and South and Southeast Asia, and thus coincide in winter with the now-established HP-H5N1 foci in those regions. The Western Hemisphere portion of the breeding distributions of these species is focused in western Alaska, but 3 species breed on the western rim of Greenland and in eastern Canada (*Charadrius hiaticula*, *Motacilla alba*, *Oenanthe oenanthe*).

**Figure 2:**
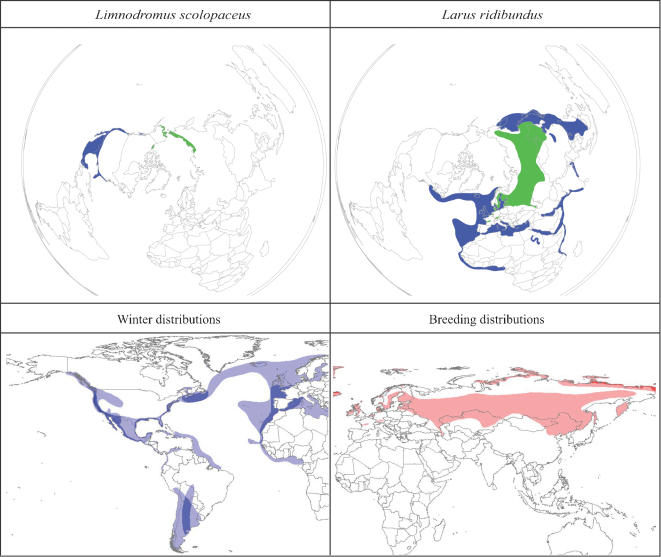
Species that breed across both hemispheres in the Holarctic, but winter at least in part in the Americas; one species (Larus ridibundus) ranges considerably farther south in Russia, extending to areas that have indeed seen HP-H5N1 outbreaks; these species winter in diverse parts of the Americas, but are focused along the Pacific, Atlantic, and Gulf coastlines of North America. The top 2 panels present examples of single species (Limnodromus scolopaceus, Larus ridibundus), whereas the lower 2 panels show species richness patterns of this set of species (see Appendix S1) in winter and in the breeding season, with color ramp indicating number of species (white = 0, darkest shade = 5 species).

In contrast, however, 10 species breed across both hemispheres in the Holarctic, but winter at least in largest part in the Americas (Fig. 2). The Eurasian portion of the breeding range of these species is mostly along the Arctic rim, but at least one species (*Larus ridibundus*) ranges considerably farther south in Russia, extending to areas that have indeed seen HP-H5N1 outbreaks (Fig. 2). These species winter in diverse parts of the Americas, but are focused along the Pacific, Atlantic, and Gulf coastlines of North America (Fig. 2); one species (*Tryngites subruficollis*; not shown) winters in southern South America.

**Figure 3:**
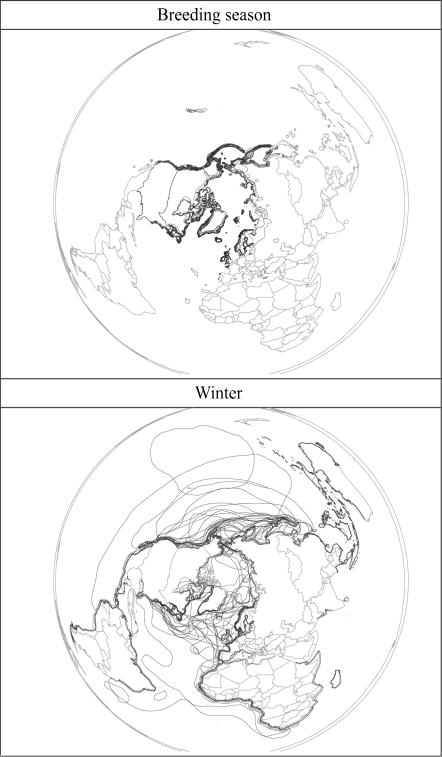
Species that are pelagic much of the year, particularly in winter. These species breed across the North Atlantic, North Pacific, and Arctic, but extend farther south in coastal and offshore areas in winter. Distributional limits for each species are shown as a black outline.

A much larger suite of species (56) is pelagic in nature, particularly in winter. These species breed across the North Atlantic and North Pacific, and extend farther south in coastal and offshore areas in winter (Fig. 3). Finally, 76 species have distributional areas in both regions in both seasons (Appendix S1)—as such, without more detailed data regarding connectivity of specific subsets of species' distributional areas (i.e., do any intercontinental movements take place among these species?), we cannot establish the degree to which intercontinental movements are or are not taking place.

## Discussion

In the paragraphs and figures above, we have explored how HP-H5N1 might arrive in North America as it spreads across Eurasia and Africa. Perhaps given the potential importance of these issues, however, several points have been controversial, and will be mentioned here.

First is the supposition that migratory birds *are* involved in regional dispersal of HP-H5N1. Some have argued that much or all of the geographic patterns of spread of HP-H5N1 is understandable based on legal or illegal movements of poultry [15]. Others have observed genetic connections among virus strains in areas connected by bird migration [16], or have tracked the general pattern of spread [21]–[23], and have arrived at the opposite conclusion—that migratory bird movements have been intimately involved in HP-H5N1 spread. We do not intend to solve this debate here—that question will clearly require extensive sampling and testing large numbers of wild birds for resolution—rather, we present scenarios of likely geographic behavior of HP-H5N1 *were migratory birds to be* a major dispersal vector.

An additional consideration is the host taxonomic distribution of HP-H5N1 infections. An almost universal dogma is that this virus infects waterfowl principally [1], [22], [23]. Clearly, as waterfowl are of large body size and tend to congregate in aquatic environments in large numbers, opportunities for transmission from individual to individual are great, and their aquatic habits apparently serve to increase the probabilities of cross-infection. In this sense, waterfowl represent a logical focus of influenza infection.

However, given the urgency of the situation, the waterfowl focus of HP-H5N1 must be considered critically so as to avoid incorrect assumptions. Detection biases are certainly involved; for instance, a small songbird dead in a forest is much less noticeable than a large, sick or dead waterbird; current surveillance programs, in fact, are focusing entirely on the waterbirds (see below). Second, the very limited data sets that are available and that present data for other than waterbirds [25] seem not to show any bias towards waterfowl and against landbirds. Finally, in studies at HP-H5N1 outbreak sites, land birds have been found to be infected with the virus. For example, an Oriental Magpie-Robin (*Copsychus saularis*) found dead in Hong Kong tested positive for H5N1 [26], suggesting that land birds *do* become infected, although their actual role in dispersal and spread of HP-H5N1 remains to be tested carefully. As such, the conclusion that waterfowl alone are the hosts of HP-H5N1 [cf. a recent review [1]] should probably be reexamined in the face of new, objective evidence. This role, whether it be large or small, does not change the conclusions of this study; rather, it only would reweight the roles of different species in HP-H5N1 dispersal and spread, but the basic patterns remain the same.

**Figure 4:**
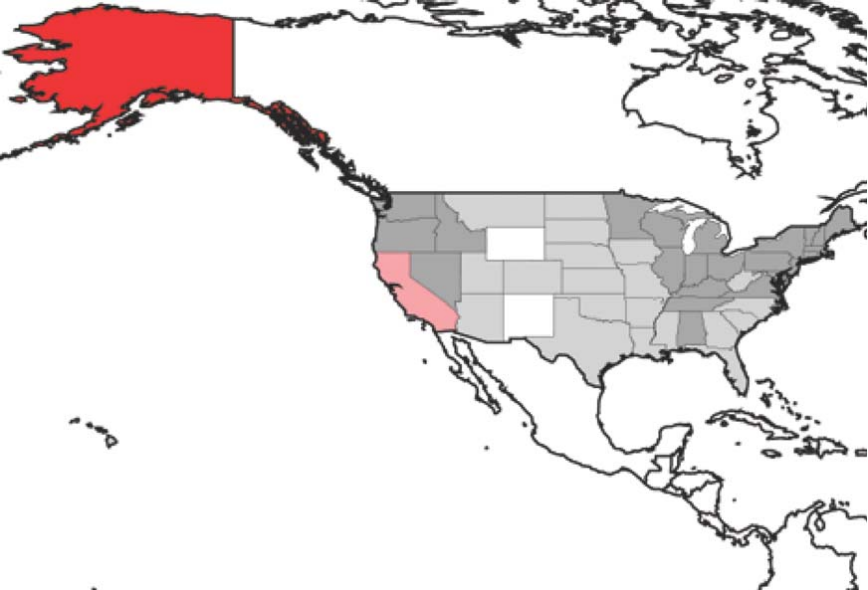
Summary of 2006 sampling (i.e., number of individuals tested) in the National HPAI Early Detection System, showing the extreme focus of efforts on Alaska (http://wildlifedisease.nbii.gov/ai/). White <10 samples, light gray 10–99 samples, medium gray 100–999 samples, light red 1000–1100 samples, red >14,000 samples.

The point of this discussion is that of reexamining the present HP-H5N1 surveillance network that has been established in North America in light of connectivity via bird migration among the northern continents. Basically, the question distills to whether the U.S. government is looking in the right places to find HP-H5N1 when or if it does arrive in North America. The National HPAI Early Detection System (HEDDS) has been established to monitor for the entry of this virus into North America, and a parallel system has been established in Canada [27]. The spatial distribution of samples tested as part of HEDDS in 2006 and its rather extreme focus on Alaska are shown in Fig. 4 (2005 sampling was parallel, if not still more concentrated in Alaska) [28]. Taking into account the Canadian sampling programs [27], the Arctic rim is better represented [27], but the coastal zones farther south remain relatively unsampled.

In the present analysis, we have shown that one component (birds breeding in North America and Eurasia and wintering in southern Eurasia or Africa) of the intercontinental migratory avifauna fits the Alaskan focus of the HEDDS surveillance. The remainder of the bird species that cross between continents, however, do not fit this mold. Rather, they have the potential to carry HP-H5N1 to the extreme northeastern rim of North America and/or farther down into North America (and even into South America), well south and east of Alaska. What is more, the primary focus of these distributional areas is coastal—Pacific, Atlantic, and even Gulf. These areas should be considered as additional—and potentially key—foci for HP-H5N1 monitoring.

More generally, this analysis points out the need for actions in response to key policy issues to be based firmly on quantitative biology and detailed analysis, rather than simply on opinion. That is, plans for HP-H5N1 early detection and monitoring should not be based on tradition and dogma (e.g., waterfowl only), but on detailed scientific preanalysis that uses the best information available in the moment to make the best decisions possible. This analysis is just a small step in that direction—other work necessary should address migratory patterns within North America, and should involve extensive testing of wild birds from HP-H5N1 infected areas to establish much more firmly the geographic, ecological, and host-taxonomic distribution of this virus.

## Methods

We reviewed summaries of species' distributions and seasonal movements in the most authoritative resource for North America [29]. We chose, in all, 157 species (both landbirds and waterbirds) that have seasonal distributions in both the Palearctic (i.e., Eurasia) and the Nearctic (North America) regions. We followed the AOU taxonomy [29], except for a few cases in which other resources [30] present updates and modifications.

We then digitized breeding and wintering ranges for each species (see Appendix S1, for a list) as polygon shapefiles in ArcGIS 9.0, based on range descriptions in recent summaries [29]–[31]; where AOU range descriptions differed with respect to Eurasian breeding status from other sources (e.g., *Tringytes subruficollis*, *Calidris bairdii*), we followed the Eurasian-specific literature resources [31]. We omitted species that do not breed or winter regularly in the Americas (e.g., *Philomachus pugnax*, *Calidris ferruginea*), although they do provide a regular influx of vagrant individuals. Species were then separated into groups of more or less comparable seasonal movements: [1] breeding in North America and Eurasia and wintering in southern Eurasia or Africa, [2] breeding in North America and Eurasia and wintering in the Americas, [3] breeding in North America and Eurasia and wintering pelagically, and [4] Holarctic, with breeding and wintering grounds in both hemispheres but no clear intercontinental movements. One species, *Larus glaucescens*, did not fit well into this schema, as it breeds in northwestern North America and winters along the Pacific coasts of both continents. Counts and manipulations of species in these four classes formed the basis for all our analyses.

## Supporting Information

Appendix S1 Summary of species included in the analysis
(0.03 MB DOC)
