# Supplementary material for: Highly Pathogenic H5N1 Avian Influenza: Entry Pathways into North America via Bird Migration
Source: PLoS One. 2007 Feb 28;2(2):e261. doi: 10.1371/journal.pone.0000261 (PMC1803015; doi:10.1371/journal.pone.0000261)
Supplement: Appendix S1 — Summary of species included in the analysis (0.03 MB DOC) [file pone.0000261.s001.doc]

**Appendix**

Summary of species included in each category of intercontinental movements. It should be noted that the differences between these categories are ones of degree, and are not black and white. *Larus glaucescens* did not fit well into the following schema, as it breeds in northwestern North America and winters along the Pacific coasts of both continents.

**Breed America or both, winter Eurasia** (14 species): *Anser brachyrhynchus*, *Anthus cervinus*, *Calidris ruficollis*, *Charadrius hiaticula*, *C. morinellus*, *Limosa lapponica*, *Luscinia svecica*, *Motacilla alba*, *M. flava*, *Oenanthe oenanthe*, *Phylloscopus borealis*, *Pluvialis fulva* (small and irregular North American wintering population), *Somateria fischeri*, and *Tringa glareola*.

**Breed Asia or both, winter at least in largest part in Americas** (10 species): *Anser caerulescens*, *Branta* *bernicla*, *Bucephala islandica*, *Calidris bairdii*, *C. mauri*, *C. pusilla*, *Grus canadensis*, *Larus ridibundus*, *Limnodromus scolopaceus*, and *Tringytes subruficollis*.

**Breed both, winter both** (76 “Holarctic” species … no direct evidence of intercontinental movements, but possible): *Accipiter gentilis*, *Aegolius funereus*, *Anas acuta*, *A. clypeata*, *A. crecca*, *A. platyrhynchos*, *A. strepera*, *Anser albifrons*, *A. canagicus*, *Anthus rubescens*, *Aquila chrysaetos*, *Ardea alba*, *Arenaria interpres*, *Asio flammeus*, *A. otus*, *Aythya marila*, *Bombycilla garrulus*, *Bubo* [= *Nyctea*] *scandiaca*, *Bubulcus ibis*, *Bucephala clangula*, *Buteo lagopus*, *Calcarius lapponicus*, *Calidris alba*, *C. alpina*, *C. canutus*, *C. maritima*, *C. melanotos*, *C. ptilocnemis*, *Carduelis flammea*, *C. hornemanni*, *Charadrius alexandrinus*, *Chen canagica*, *Chlidonias niger*, *Circus cyaneus*, *Clangula hyemalis*, *Cygnus columbianus*, *Eremophila alpestris*, *Falco columbarius*, *F. peregrinus*, *Gallinago gallinago*, *Gallinula chloropus*, *Hirundo rustica*, *Histrionicus histrionicus*, *Lagopus lagopus*, *L. mutus*, *Lanius excubitor*, *Larus argentatus*, *L. canus*, *L. minutus*, *Loxia curvirostra*, *L. leucoptera*, *Melanitta fuscata*, *M. nigra*, *Mergus merganser*, *M. serrator*, *Nycticorax nycticorax*, *Pandion haliaetus*, *Phalaropus fulicaria*, *P. lobatus*, *Pinicola enucleator*, *Plectrophenax nivalis*, *Plegadis falcinellus*, *Pluvialis squatarola*, *Podiceps auritus*, *P. grisigena*, *P. nigricollis*, *Riparia riparia*, *Somateria mollissima*, *S. spectabilis*, *Sterna caspia*, *S. nilotica*, *S. sandvicensis*, *Strix nebulosa*, *Surnia ulula*, *Tringa incanus*, and *Troglodytes troglodytes*.

**Breed Holarctic, winter pelagic or coastally in northern oceans** (56 “pelagic” species): *Aethia cristatella*, *A. pusilla*, *A. psittacula*, *A. pygmaea*, *Alca torda*, *Alle alle*, *Brachyrhamphus brevirostris*, *B. perdix*, *Calonectris diomedea*, *Catharacta skua*, *Cepphus columba*, *C. grylle*, *Cerorhinca monocerata*, *Cyclorrhynchus psittaculus*, *Diomedia albatrus*, *D. immutabilis*, *D. nigriceps*, *Fratercula arctica*, *F. cirrhata*, *F. corniculata*, *Fregata minor*, *Fulmarus glacialis*, *Gavia adamsii*, *G. arctica*, *G. immer*, *G. pacifica*, *G. stellata*, *Larus glaucoides*, *L. hyperboreus*, *L. marinus*, *Morus bassanus*, *Numenius phaeopus*, *N. tahitiensis*, *Oceanodroma furcata*, *O. leucorhoa*, *Pagophila eburnea*, *Phalacrocorax carbo*, *P. pelagicus*, *P. urile*, *Polysticta stelleri*, *Puffinus gravis*, *P. griseus*, *Puffinus puffinus*, *Rissa tridactyla*, *Stercorarius longicaudus*, *S. parasiticus*, *S. pomarinus*, *Sterna aleutica*, *S. dougallii*, *S. fuscata*, *S. hirundo*, *S. paradisaea*, *Synthliboramphus antiquus*, *Uria aalge*, *U. lomvia*, and *Xema sabini*.
